# Combined phylogeny and neighborhood analysis of the evolution of the ABC transporters conferring multiple drug resistance in hemiascomycete yeasts

**DOI:** 10.1186/1471-2164-10-459

**Published:** 2009-10-01

**Authors:** Marie-Line Seret, Julie F Diffels, André Goffeau, Philippe V Baret

**Affiliations:** 1 Unité de Génétique (GENA), Université Catholique de Louvain, Louvain-la-Neuve, Belgium; 2 Unité de Biochimie Physiologique (FYSA), Institut des Sciences de la Vie (ISV), Université Catholique de Louvain, Louvain-la-Neuve, Belgium

## Abstract

**Background:**

Pleiotropic Drug Resistant transporters (PDR) are members of the ATP-Binding Cassette (ABC) subfamily which export antifungals and other xenobiotics in fungi and plants. This subfamily of transmembrane transporters has nine known members in *Saccharomyces cerevisiae*.

We have analyzed the complex evolution of the pleiotropic drug resistance proteins (Pdrp) subfamily where gene duplications and deletions occur independently in individual genomes.

This study was carried out on 62 Pdrp from nine hemiascomycetous species, seven of which span 6 of the 14 clades of the *Saccharomyces *complex while the two others species, *Debaryomyces hansenii *and *Yarrowia lipolytica*, are further apart from an evolutive point of view.

**Results:**

Combined phylogenetic and neighborhood analyses enabled us to identify five Pdrp clusters in the *Saccharomyces *complex. Three of them comprise orthologs of the Pdrp *sensu stricto*, Pdr5p, Pdr10p, Pdr12p, Pdr15p, Snq2p and YNR070wp. The evolutive pathway of the orthologs of *Snq2 *and *YNR070w *is particularly complex due to a tandem gene array in *Eremothecium gossypii, Kluyveromyces lactis *and *Saccharomyces (Lachancea) kluyveri*. This pathway and different cases of duplications and deletions were clarified by using a neighborhood analysis based on synteny.

For the two distant species, *Yarrowia lipolytica *and *Debaryomyces hansenii*, no neighborhood evidence is available for these clusters and many homologs of *Pdr5 *and *Pdr15 *are phylogenetically assigned to species-based clusters.

Two other clusters comprise the orthologs of the *sensu lato *Pdrp, Aus1p/Pdr11p and YOL075cp respectively. The evolutionary pathway of these clusters is simpler. Nevertheless, orthologs of these genes are missing in some species.

**Conclusion:**

Numerous duplications were traced among the Hemiascomycetous Pdrp studied. The role of the Whole Genome Duplication (WGD) is sorted out and our analyses confirm the common ancestrality of Pdr5p and Pdr15p. A tandem gene array is observed in *Eremothecium gossypii*. One of the copies is the ortholog of *Snq2 *while the other one is lost in the post-WGD species.

The neighborhood analysis provides an efficient method to trace the history of genes and disentangle the orthology and paralogy relationships.

## Background

The phenotype for pleiotropic drug resistance (PDR) in *Saccharomyces cerevisiae *(SACE) was discovered in 1973 by RANK and BECH-HANSEN[1], who reported that single gene mutations were responsible for resistance to multiple drugs of different chemical structures and different targets. The genetic and molecular mapping revealed that these mutations are located in the transcription factors Pdr1p and Pdr3p [2-4] and increased the expression of a series of target genes [5] including those encoding the ABC efflux pumps Pdr5p [6-8], Snq2p [9], Pdr10p and Pdr15p [10,11] or Pdr11p [10].

Analysis of the full genome sequence of *Saccharomyces cerevisiae *[12] revealed that these transporters are members of a large phylogenetic subfamily of ABC transporters named Pdrp (for pleiotropic drug resistance proteins), where nuclear binding folds (NBF) alternate with domains comprising six predicted transmembrane spans (TMS) to form a NBF-TMS-NBF-TMS pattern [13]. NBF includes a distinctive series of amino acid sequence motifs, including Walker A, Walker B and ABC signatures involved in ATP binding and hydrolysis.

The inventory of all full-sized *Saccharomyces cerevisiae *ABC transporters reveals six Pdrp *sensu stricto *namely: Pdr5p, Pdr10p, Pdr12p, Pdr15p, Snq2p and YNR070wp [13], characterized by three criteria: (1) The alternation NBF-TMS-NBF-TMS, which is different from the classical TMS-NBD-TMS-NBD topology reported for the mammalian multidrug resistance MDR and MRP drug efflux pumps [14,15]; (2) The presence of a cysteine residue (C) instead of the lysine residue (K) in N-terminal Walker A motifs as well as the specific NVEQ motif in the C-terminal ABC signature [6,13]; (3) The efflux of multiple drugs [16-18].

The structural determinants for the broad substrate specificity of Pdrp pumps have not yet been identified even though dozens of publications have reported hundreds of amphiphilic substrates for Pdr5p and Snq2p. These two pumps share many substrates even though some drugs are preferentially handled by either one or the other [17,19]. In contrast, Pdr12p exhibits clear substrate specificity for short chain weak organic acids [20].

Fully sequenced Ascomycota and Basidiomycota were all examined and found to contain several Pdr proteins [21] but their evolutionary lineage is unclear. A previous phylogenetic classification of Pdrp from five species spanning the full Hemiascomycetes phylum identified seven subclusters [22]. GBELSKA*et al*. [23] showed that in contrast to the other ABC subfamilies which remain as single copy in each species during evolution, a series of gene duplications occurs independently in the PDR subfamily in five hemiascomycetous species. GAUR*et al*. [24] compared the NBF domains of *S. cerevisiae *and *Candida albicans *Pdrp. They concluded that *C. albicans *contains a cluster of four proteins homologs to Pdr5p and a single protein homolog to Snq2p.

In addition to the Pdrp clusters *sensu stricto*, the *S. cerevisiae *genome contains two other ABC phylogenetic clusters exhibiting the alternation of NBF-TMS-NBF-TMS domains [13]. One cluster comprising Aus1p and Pdr11p are sterol-influx pumps [25] while another cluster comprising YOL075cp is of undetermined function. As the members of these two phylogenetic clusters show neither drug efflux properties nor the K/C substitution in the Walker A1 motif, we classified them as Pdrp *sensu lato*.

The aim of the present paper is to trace the evolution of the Pdrp subfamily in nine species belonging to the *Hemiascomycetes *phylum, believed to have separated from the filamentous fungi 300 to 400 million years ago [26,27]. Seven of these species span the 14 clades constituting the *Saccharomyces *complex [28]. Among those, three novel genome sequences from the *Lachancea *or *Zygosaccharomyces *clades were analyzed: *Saccharomyces (Lachancea) kluyveri *(SAKL), *Zygosaccharomyces rouxii *(ZYRO) *and Kluyveromyces (Lachancea) thermotolerans *(KLTH) [29]. Two hemiascomycetous sequences of older lineage; *Debaryomyces hansenii *(DEHA) and *Yarrowia lipolytica *(YALI) were also explored. An exhaustive database of all the chromosomal neighbors of the PDR genes from all nine hemiascomycetous species was developed (SERET*et al*., in preparation) and allowed us to carry out combined phylogenetic and neighborhood analysis. Gene neighborhood analysis has been pioneered by BYRNE and WOLFE[30] for tracing the ancestral gene blocks produced by the Whole Genome Duplication (WGD). We will illustrate the extent and the limits of the information provided by analysis of gene neighborhood data for deciphering the evolution of the large Pdrp subfamily in addition to classical phylogenetic analysis based only on amino acid sequence similarity.

## Results

**Figure 1:**
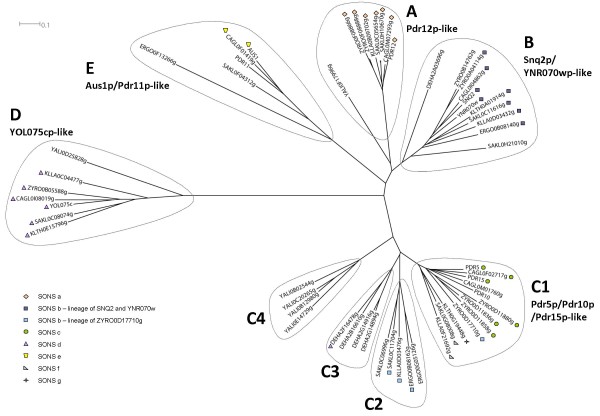
**Phylogenetic tree of the 56 PDR produced by Neighbor-Joining**. The tree shows five clusters identified by a capital letter as well as by the name of their *S. cerevisiae *members. The symbols identify genes belonging to the same Subset of Orthologs by Neighborhood and Similarity.

The Pdrp transporters are a well characterized family of genes implied in drug resistance. All genes share typical features, such as Walker A and B and an ABC signature, and are very similar in terms of sequence. In the Génolevures database, the family GL3C0025 which contains all SACE Pdrp comprises 62 members (Table 1) of which 6 are considered as pseudogenes or fragments (Additional file 1). The phylogenetic tree in Figure 1, based exclusively on full-length proteins to improve multiple alignment, shows that the 56 full-Pdr proteins of the GL3C0025 family (Additional file 2) branch into five clusters labeled A to E. Each cluster is named according to its *S. cerevisiae *member(s): A, the cluster Pdr12p (8 members); B, the cluster Snq2p/YNR070wp (11 members); C, the cluster Pdr5p/10p/15p (25 members) which ramifies into 4 subclusters labeled C1, C2, C3, C4; D, the cluster YOL075cp (7 members); E, the cluster Aus1p/Pdr11p (5 members).

**Table 1:** The members of the GL03C0025 family comprising all SACE Pdrp

				**Homologs to**			
**Species**	**Acronym**	**Clade**^1^	**PDR sensu stricto**	**YOL75c**	**to AUS1**	**Full size ABC transporters**	**Number of *fragments***
*Saccharomyces cerevisiae*	SACE	1	6	1	2	9	-
*Candida glabrata*	CAGL	4	4	1	1	6	-
*Zygosaccharomyces rouxii*	ZYRO	7	8	1	-	9	-
*Kluyveromyces thermotolerans*	KLTH	10	2	1	-	3	2
*Saccharomyces kluyveri*	SAKL	10	7	1	1	9	1
*Kluyveromyces lactis*	KLLA	11	4	1	-	5	-
*Eremothecium gossypii*	ERGO	12	3	-	1	3	-
*Debaryomyces hansenii*	DEHA	-	5	-	-	6	3
*Yarrowia lipolytica*	YALI	-	5	1	-	6	-

**Total**			**44**	**7**	**5**	**56**	**6**

^1 ^according to KURTZMAN[28]

**Figure 2:**
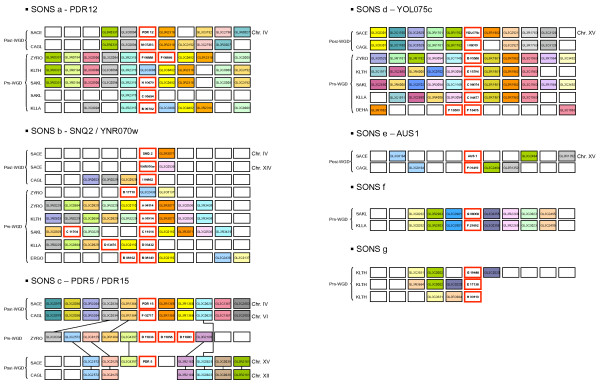
**Manually curated Subsets of Orthologs by Neighborhood and Similarity of Pdrp**. SONS a: Orthologs of *Pdr12 *(cluster A). SONS b: Orthologs of *Snq2 *and *YNR070w *(cluster B). SONS c: Orthologs of *Pdr5 *and *Pdr15 *(cluster C). SONS d: Orthologs of *YOL075c *(cluster D). SONS e: Orthologs of *Aus1 *(cluster E). SONS f: Subset of Orthologs by Neighborhood and Similarity consisting of *SAKL0G08008g *and *KLLA0F21692g *(cluster C). SONS g: Subset of Orthologs by Neighborhood and Similarity made up of *KLTH0G19448g *(cluster C) and the two telomeric fragments *KLTH0E17138g *and *KLTH0H00110g*. Each box framed in red represents a PDR. Adjacent boxes represent the PDR neighbors. Homologous neighbors, based on Génolevures families, are highlighted in the same color. These SONS were calculated with 15 neighbors on each side, but the representation was truncated to 5 neighbors for the sake of clarity.

Neighborhood analysis (Additional file 3) was carried out to delineate Subsets of Orthologs by Neighborhood and Similarity (SONS). Seven SONS labeled "a" to "g" (Figure 2) provide information on the evolution of 40 proteins displaying informative neighborhood among the 62 PDR from the GL3C0025 family.

For most genes, the phylogenetic clusters are confirmed by the neighborhood analysis. But in some cases, divergences are observed. In the next sections, each phylogenetic cluster of genes is discussed in an evolutionary perspective integrating the evidence from the neighborhood analysis.

### Cluster A (Pdr12p)

This cluster comprises 8 members in SACE, CAGL (*Candida glabrata*), ZYRO, SAKL, KLLA (*Kluyveromyces lactis*) and YALI (Figure 1). No full-Pdrp orthologs are detected in KLTH, even though the neighborhood is well maintained on chromosome E, ERGO (*Eremothecium gossypii*) and DEHA. The neighborhood analysis (Figure 2 SONS a) shows a clear connection (9 neighbors) between *SAKL0H10670g *and the PDR belonging to the other species, except YALI. A second PDR copy is present in SAKL on chromosome C. *SAKL0CO5654g *only shares one neighbor, belonging to the GL3R2315 family. The very high amino acid identity (98%, 17 substitutions) between the two paralogs suggests a very recent duplication. A tandem gene array occurred in ZYRO where the two copies diverged a little more (87% amino acid identity). None of these duplications (on different chromosomes in SAKL and within the same chromosome in ZYRO) were maintained in other species. Pdr12p and Snq2p are in closely related clusters as they share a high similarity rate. Despite this proximity of sequence, neighborhood evidence shows that they evolved separately, at least since the speciation time of KLLA.

### Cluster B (Snq2p/YNR070wp)

**Figure 3:**
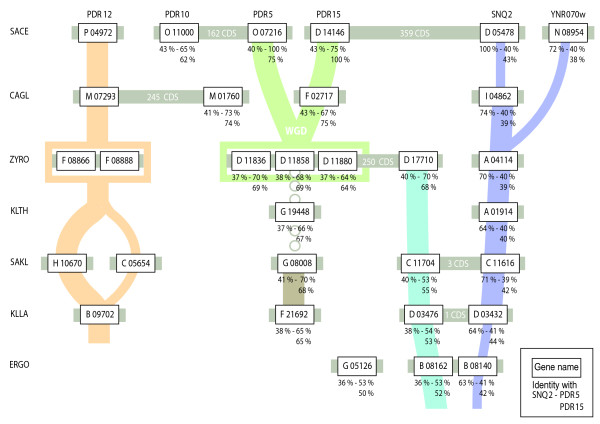
**Relationships between the PDR *sensu stricto *based on the neighborhood analysis**. Each box represents a PDR gene. Grey lines join PDR genes located on the same chromosome. When non-adjacent, the number of coding sequences between two PDR genes is noted in white. Note that *SAKL0C05654g, SAKL0C11616g *and *SAKL0C11704g *are on the same chromosome. Shared neighborhoods are represented by connectors whose colors match those of Figure 1. Two colors were used for SONS b in order to distinguish the lineage of *ZYRO0D17710g *and the one of *Snq2 *and *YNR070w*. A large connector links PDR genes sharing more than one common neighbor while a thin connector links PDR genes sharing one neighbor only. Dotted connectors link genes whose relationship is based on phylogenic evidence only. For the story leading to *Pdr5/15 *and *Snq2*, the percentage of identity with their homologs in the other species is given. The label WGD stands for Whole Genome Duplication. Our representation does not imply that the present ERGO species is the ancestor of all the other species we analyzed.

The phylogenetic Snq2p cluster comprises 11 members that are represented in all the examined species except YALI. A shared neighborhood is present from ERGO to SACE (Figure 2 SONS b). It comprises a single ortholog of Snq2p in each species except in SACE where YNR070wp is a paralog of Snq2p. Moreover, neighborhood analysis shows an apparent tandem gene array in ERGO chromosome B. However, the two copies are different (38% amino acid identity). ERGO0B8140p has 63% identity to Snq2p and only 41% identity to Pdr5p (Figure 3). In contrast, ERGO0B8162p has a higher amino acid identity to Pdr5p (53%) than to Snq2p (36%). A similar heterologous tandem in a similar neighborhood is observed for KLLA and SAKL with, however, the insertion of an increasing number of genes between the two members of the repeat. ZYRO also presents two genes in this neighborhood but they are no longer in tandem gene array. It is noteworthy that KLTH only has one gene, an ortholog of *Snq2*, in this neighborhood.

The phylogenetic tree in Figure 1 also shows that paralogs of *Snq2 *are observed in SACE (*YNR070w*), ZYRO (*ZYRO0B14762g*) and SAKL (*SAKL0H21010g*) in non-shared chromosomal environments. *YNR070w *and *ZYRO0B14762g *probably derive from a species specific duplication event. The case of *SAKL0H21010g *suggests a duplication followed by a rapid evolution of the copy. In terms of identity, the *DEHA0A03696g *gene, which does not share any neighbor with any *Snq2 *or *Pdr12*'s homologs, is closer to Snq2p (56%) than to Pdr12p (44%).

### Cluster C (Pdr5p/10p/15p)

The phylogenetic C cluster contains 25 members including the SACE Pdr5p/10p/15p. Figure 2 SONS c shows that in the pre-WGD species ZYRO, three copies of *Pdr5/15 *orthologs (*ZYRO0D11836g, ZYRO0D11858g, ZYRO0D11880g*) are organized in tandem on chromosome D. The triplicated ortholog copies of *Pdr5/15 *from ZYRO are inserted in a neighborhood that can be traced back in KLLA, SAKL and KLTH but none of these chromosomal environments contain a *Pdr5/15 *homolog. However, in each of these species a *Pdr5/15*-like copy is present in a neighborhood different from that of the ZYRO triplicate. The KLTH, SAKL and KLLA copies show a strong phylogenetic link with Pdr5p/15p: nearly 67% amino acid identity for KLTH0G19448p, nearly 70% amino acid identity for SAKL0C08008p and nearly 65% amino acid identity for KLLA0F21692p. *KLLA0F21692g *and *SAKL0C08008g *share a common neighborhood (see Figure 2, SONS f) which is not shared with *KLTH0G19448g *(see Figure 2, SONS g).

The ancestor of the ZYRO copies was duplicated by the WGD and produced the ohnologs *Pdr5 *and *Pdr15 *[30]. In fact, *Pdr15*, which is located on chromosome D, possesses two common neighbors with ZYRO while *Pdr5*, located on chromosome F, shares five common neighbors with ZYRO. Only the *Pdr15 *copy was retained in CAGL in this neighborhood. A second PDR, *CAGL0M01760g*, with a high amino acid identity with Pdr5p/15p is present in CAGL in another neighborhood.

Pdr10p shows high amino acid identity to Pdr5p/15p (65% and 62% respectively). Segment alignment reveals that Pdr10p can be differentiated from other SACE Pdrp by a 22 amino acid insert (SDAAIMGNDKTVAKEHYSSPSS), located at the intersection of the two half PDR-regions (NBF-TMS-insert-NBF-TMS). This marker means that Pdr10p can be detected in *S. cerevisiae, Saccharomyces bayanus, Saccharomyces paradoxus *and *Saccharomyces mikatae *but no ortholog was found in CAGL and in the pre-WGD species (data not shown, inferred from Broad Institute [31]). The event that gave rise to Pdr10p is thus post-WGD.

In addition to the major subcluster C1 (Figure 1) comprising Pdr5/10/15p, cluster C contains three additional phylogenetic subclusters. Two of these subclusters show a spectacular species-based clustering. Indeed, very similar proteins tend to cluster with proteins belonging to the same species rather than with orthologs in other species. One of the subcluster (C3) comprises five DEHA members. The neighborhood analysis suggests a putative link between DEHA2F16478g and the orthologs of YOL075c. The other subcluster (C4) comprises four YALI members. Neighborhood analysis of this subcluster is not conclusive but phenotype analysis reveals that one of them (YALI0C20265p) is an azole efflux transporter with properties similar to those of Pdr5p/Snq2p [32] while another (YALI0E14729p) has the unique property to export C16 hydrocarbon residues [32].

The last subcluster (C2) contains five members:*SAKL0C06996g*, *SAKL0C11704g, KLLA0D03476g *as well as *ERGO0B08162g *and *ERGO0G05126g *which are very similar (73% amino acid identity). Three of them, *ERGO0B08162g, KLLA0D03476g *and *SAKL0C11704g *are in tandem gene array. Interestingly, they belong to the C cluster (Pdr5p/10p/15p) when using phylogenetic criteria but share some neighbors with the B cluster (Snq2p). This is also the case of *ZYRO0D17710g *of the subcluster C1.

### Cluster D (YOL075cp)

This cluster comprises a total of 7 members; one per species except in DEHA and ERGO. A spectacular shared neighborhood presence is observed for the orthologs of *YOL075c *(see Figure 2, SONS d). Eight common neighbors are, for instance, identified in the post-WGD species SACE and CAGL, four common neighbors are identified in the pre-WGD species and four common neighbors link ZYRO, one of the pre-WGD species to the post-WGD species. The YALI homologue (YALI0D25828p) has no conclusive neighborhood but it exhibits high amino acid identity (41%) to the SACE YOL075cp. This suggests that all hemiascomycetous orthologs of *YOL075c *originate from an ancestor existent prior to the speciation event of YALI.

The neighborhood analysis links a tandem gene array made up of *DEHA2F16478g *and the fragment *DEHA2F16500g *to cluster D. However, the *DEHA2F16478g *is classed in subcluster C3 by phylogeny and does not display the specific motifs of YOL075cp orthologs.

### Cluster E (Aus1p/Pdr11p)

This cluster comprises members of four species only: SACE, CAGL, SAKL and ERGO. Using neighborhood analysis, an ortholog of Aus1p was only identified in CAGL. There is no evidence that the two SACE paralogs, Aus1p on chromosome O and Pdr11p on chromosome I, issue from the WGD as there is no shared neighborhood with pre-WGD species. In SACE, both Aus1p and Pdr11p are essential for anaerobic growth as they contribute to the uptake of ergosterol under conditions which prevent the aerobic synthesis of sterols [25]. The sterol transporter function was recently demonstrated for the CAGL ortholog of Aus1p [33]. The two characteristic Pdrp motifs, N-terminal Walker A and the C-terminal ABC signature, remain, but only partially (Additional file 1).

### Fragments

Six fragments of 72 to 1099 amino acids belonging to the GL3C0025 protein family have been identified. Despite their subtelomeric location, that we define as the last 30 kb of each chromosome following FAIRHEAD and DUJON [34], the two fragments *KLTH0E17138g *and *KLTH0H00110g *share the neighborhood of *KLTH0G19448g*, the ortholog of *Pdr5/15*, which may suggest duplications followed by pseudogenization. Because phylogenetic analysis of fragments may be error-prone, their evolutionary significance will not be analyzed.

## Discussion

Orthologous PDR transporters with typical NBF-TMS-NBF-TMS topology are found in all higher fungi such as *S. cerevisiae *[6,13], the hemiascomycetous human pathogen *C. albicans *[35], *Euascomycetes *[36], *Archiascomycetes *[37,38], *Basidiomycetes *[39] and all plants [40] including *Arabidopsis thaliana *[41] and *Nicotiana plumbaginifolia *[42]. In contrast, the PDR topology has not been detected in protozoa [43] or chordates [44] where multiple drug resistance results from the overexpression of MDR/MRP efflux transporters of opposite TMS-NBF-TMS-NBF topology.

Our analysis of 56 full-sized Pdrp from 9 different Hemiascomycetes shows that the Génolevures subfamily GL3C0025 [29] is subdivided into five phylogenetic clusters labeled A to E (Figure 1). Each of these clusters can be characterized by its SACE members originally identified by DECOTTIGNIES and GOFFEAU[13]. Within the phylogenetic *Saccharomyces *complex defined by KURTZMAN[28], a total of 40 Pdrp homologs sharing significant chromosomal neighborhood were classified in seven SONS, labeled "a" to "g" (Figure 2), in which phylogenetic and neighborhood data are combined.

It now appears that the Pdrp *sensu stricto *phenotype can be allocated only to cluster A (Pdr12p), to cluster B (Snq2p/YNR070wp) and to cluster C (Pdr5p/10p/15p), given that all three share not only the NBF-TMS-NBF-TMS topology but also the two additional Pdrp traits: efflux drug pumping and typical Walker A1 and ABC Signature 1 motifs.

On the other hand, no experimental information is available concerning the unknown substrates of the members of cluster D (YOL075cp), while the SACE members of cluster E (Aus1p/Pdr11p) are clearly sterol influx pumps [25]. Moreover, as neither cluster D nor cluster E contains members with the typical Pdrp motifs, they may be considered to be Pdrp *sensu lato*.

Our combined phylogenetic and neighborhood analyses support the evolutionary pattern illustrated in Figure 3 for the Pdrp *sensu stricto *of the *Saccharomyces *complex [28].

Pdr12p is a Pdr *sensu stricto*, according to our three criteria. Its origin remains questionable as no Pdr12p neighbors are shared either with Pdr5p/15p or Snq2p. In SACE, the function of Pdr12p, which effluxes food preservatives such as short chain weak acids [20], is different from that of Snq2p and Pdr5p which share the function to efflux a series of antifungal azoles and other hydrophobic substrates [17]. Nevertheless, the SACE Pdr12p sequence is closer to SACE Snq2p (46%) than to SACE Pdr5p (38%). This may suggest a common origin of both Pdr12p and Snq2p clusters. The sequence analysis of new yeast species, phylogenetically situated between DEHA and ERGO, should make it possible to disentangle the exact evolution lineage.

The unlinked ancestor of Pdr5p/15p ohnologs of KLTH jumped and triplicated on ZYRO chromosome D. An ancestor of the three copies, already in this neighborhood, was duplicated through WGD and produced Pdr5p and Pdr15p in all *Saccharomycetes*.

The phylogenetic tree of the Pdr5p/15p clusters shows several subclusters (C3 and C4) which aggregates members belonging to specific species such as DEHA or YALI. These are cases where species-based clustering is suspected of hindering a functional inference based on sequence similarity only. For example, the subcluster C4 contains four YALI members belonging to four different chromosomal fragments in unshared environments. Despite being inside the same subcluster, theses genes seem to have very different functions. One of them, *YALI0E14729g*, exhibits the function of alkane efflux, unique as yet, while another, *YALI0C20265g*, seems to control the classic azole resistance function of the SACE Pdr5p/15p pumps.

The evolutionary patterns of the Pdrp *sensu lato *are simpler and totally independent from that of the Pdr *sensu stricto*. The members of Pdr *sensu lato *Aus1p/Pdr11p cluster are present in four different species only: ERGO, SAKL, CAGL and SACE. These genes may be lost in other species. Indeed, sterol influx pumps are essential only under anaerobiosis conditions. However, no clear relation between the presence of homologs of Aus1p or Pdr11p sterol-influx pumps and facultative anaerobic growth could be detected among the species composing the *Saccharomyces *complex [45].

The members of the Pdr *sensu lato YOL075c *cluster are of unknown function and belong to species of both ancestral clade (*YALI0D25828g*) and recent clades. No homolog of *YOL075c *is found in DEHA and ERGO. Extensive neighborhood preservation is observed for all *Kluyveromycetes *and *Saccharomycetes *species. Even though the substrates of this cluster of putative transporters are unknown, its continued presence in many pre- and post-WGD species from the *Saccharomyces *complex indicates that these gene products exert an important physiological function. This function and the subcellular localization of the transporter have not yet been determined. Curiously all members of the *YOL075c *SONS have at least 100 less amino acid residues (1247 to 1328) than all other members of the GL3C0025 family. Moreover, the cysteine of SGCT motif of the N-Walker A is replaced by the "classic" lysine residue and the NVEQ motif of the C-Walker Signature is replaced by SGGE (Additional file 1).

In three cases only, we observed a divergence between the neighborhood and phylogenetic analyses. *ERGO0B08162g, KLLA0D03476g *and *SAKL0C11704g *are allocated to the C cluster (Pdr5p/15p) by phylogeny and to the SONS b (Snq2p/YNR070wp) by neighborhood. This divergence can be explained by the fact that they belong to a tandem gene array in which a copy has been subjected to neofunctionalization. In another case, *ZYRO0D17710g *(subcluster C1) is allocated to SONS b (Snq2p/YNR070wp) by neighborhood. Its high amino acid similarity with Pdr5p (70%) and significant difference of sequence with *SAKL0C11704g *and *ZYRO0A04114g *suggest an evolution based on gene conversion with one of the member of the *ZYRO0D11836g, ZYRO0D11858g *and *ZYRO0D11880g *tandem gene array. Finally, the divergence observed for *DEHA2F16478g *(subcluster C3 and SONS d) may be linked to a species-based clustering of DEHA genes within the subcluster C3.

## Conclusion

In order to assess the evolution of genes using a neighbourhood analysis, we considered four more species in addition to those examined in the study by Gbelska *et al*. [23]. Our classification is consistent for the 24 genes in the five species we share with Gbelska *et al*. Moreover, the additional species enabled us to follow more accurately the evolution of the chromosomal environments of the Pdrp and to distinguish between paralogous and orthologous status, and, in so doing, clarify the fate of some ERGO and KLLA genes. We found that some orthology relationships are confused by SYNERGY [23,46] (see Additional file 4). Our analysis converged with the Yeast Gene Order Browser [30], except for the orthology relationship of the two members of the KLLA tandem gene array which are inverted in the YGOB.

We demonstrate that, combined with phylogenetic analysis, neighborhood analysis allows us to trace back the detailed evolutionary history of most members of the phylogenetic subfamilies of Pdrp *sensu stricto *from SACE, CAGL, ZYRO, KLTH, SAKL, KLLA and ERGO. These species span 6 out of the 14 clades from the phylogenic *Saccharomyces *complex [28] that may have separated from the other yeasts around 150 million years ago. We may therefore conclude that Pdr5p and Pdr15p issued from a single ancestral gene present before the WGD. In contrast, both Pdr10p (a Pdr5p paralog) and YNR070wp (a Snq2p paralog) originated from independent post-WGD duplication events.

We show that for a large subfamily such as Pdrp, whose members share high amino acid sequence similarity, neighborhood analysis can be used to trace the duplicates from pre-WGD or post-WGD and to describe in detail the tandem or repeat duplications and the gains, loss or transposition of gene copies.

## Methods

### Pdrp

Protein sequences from *Saccharomyces cerevisiae *(SACE), *Candida glabrata *(CAGL), *Zygosaccharomyces rouxii *(ZYRO), *Kluyveromyces thermotolerans *(KLTH), *Saccharomyces kluyveri *(SAKL), *Kluyveromyces lactis *(KLLA), *Eremothecium gossypii *(ERGO), *Debaryomyces hansenii *(DEHA) and *Yarrowia lipolytica *(YALI) were retrieved from the Génolevures database in July 2007. A bioinformatics analysis based on BLAST and Smith-Waterman alignments, clustering and Condorcet election, was applied to the Génolevures data set by NIKOLSKI and SHERMAN[47] in order to define consensus families. All SACE Pdrp were found to belong to a single family, the GLC2004, recently relabeled GL3C0025. This family comprises 62 members (Table 1). Six peptides of 72 to 1099 amino acid residues were considered as fragments, leaving 56 full-sized members which were further analyzed. Genes are labeled according to the Génolevures nomenclature [48].

### Principle of assignation to SONS

Within the Génolevures GLC3C0025 family, the Subsets of Orthologs by Neighborhood and Similarity (SONS) were identified as schematized in Additional files 2 and 3. Fifteen neighbor genes were retrieved on each side of the 62 PDR (called "query gene"). Each of these 30 genes is assigned to a Génolevures family. As family assignment is based on similarity criteria, neighbor genes belonging to the same GL family were considered orthologs by similarity. Two PDR query genes, sharing at least one neighbor being classified in the same GL family, were regarded as belonging to the same SONS. Indeed, in this case, the orthology based on similarity evidences is confirmed by the neighborhood information. Out of the 62 Pdrp, 40 show informative neighborhoods. Due to the complexity of the PDR evolutionary history, the SONS were manually curated to remove some weak links between chromosomal environments. The curated output of the SONS analysis is presented in Figure 2. For the sake of clarity, the representation is limited to five neighbor genes on each side of the query PDR genes. In fact, no conclusive neighborhood could be identified in the DEHA or YALI species. Therefore all neighbor analyses of Figure 2 excluded DEHA and YALI and were restricted to the seven species we examined from the *Saccharomycetes *complex defined by KURTZMAN [28], except one DEHA gene in SONS d.

### Motifs

The Pdrp sequences were aligned with ClustalW [49] on  and visualized with Jalview [50]. The N- Walker A and C-terminal ABC signature motifs were identified based on alignments with the motifs defined by DECOTTIGNIES and GOFFEAU[13] for the Pdrp of *S. cerevisiae*. Identity was calculated after removing the filter for low complexity.

### Phylogenetic Tree

Multiple alignment was calculated by MUSCLE [51]. The phylogenetic tree in Figure 1 was constructed using PROTDIST and NEIGHBOR in the PHYLIP suite [52]. The use of Maximum likelihood (PROTML) or parsimony approach gave identical results. Only NEIGHBOR JOINING results are, therefore, presented. Representation of the phylogenetic tree made use of the Dendroscope application [53].

## Authors' contributions

M-LS and JD designed and developed a computer program to identify SONS. M-LS did the manual curation of SONS and the preparation of figures. JD prepared the tables. AG identified the PDR motifs and PB did the phylogenetic study. M-LS and JD drafted manuscripts with revisions from AG and PB. All authors read and approved the final manuscript edited by M-LS.

## Supplementary Material

Additional file 1 **List of Pdrp features included in this paper**. Sequences of *S. cerevisiae *[12], *C. glabrata, K. lactis, D. hansenii, Y. lipolytica *[54] and *E. gossypii *[55] were previously published. *Z. rouxii *and *K. thermotolerans *have both been sequenced by Génoscope, and *S. kluyveri *was sequenced by the Washington University Genome Sequencing Centre. The three new genomes ZYRO, KLTH, SAKL, plus KLLA have been entirely annotated and manually curated by the Génolevures consortium [29]. ERGO annotation is by GATTIKER[56]. Click here for file

Additional file 2 **Schema of analysis tools and databases**. Rectangles represent databases and results while parallelograms represent tools used to obtain them. Click here for file

Additional file 3 **Steps of the Identification of Orthologs by Neighborhood and Similarity method**. (1) The building of SONS (Subset of Orthologs defined by Neighborhood and Similarity) begins by taking a family of genes whose translation products show sequence similarity for example, the family GL3C0025. (2) For each query gene of this family we identify 15 genes, which we call neighbors, on each side and especially the family to which these neighbors belong. (3) Two query genes whose translation products belong to the same family (homologs by similarity) are members of the same SONS if they share at least one pair of neighbors that are also homologous to each other by similarity. These neighbors are highlighted in the same color. In the example, YOL075c shares some neighbors with the query gene of CAGL and has one neighbor in common with the query gene of KLTH. CAGL has no gene in common with KLTH, however YOL075c must be in the same SONS as the query genes of CAGL and KLTH. In consequence, the three query genes are clustered in the same SONS. Homologs that do not share a pair of homologous neighbors are separated into two distinct SONS. (4) See Figure 2 SONS d for the complete SONS of YOL075c. Click here for file

Additional file 4 **Relationships between the Pdrp *sensu stricto *based on the neighborhood analysis: comparison with YGOB and SYNERGY**. Each box represents a PDR. Grey lines join PDR genes located on the same chromosome. When non-adjacent, the number of coding sequences between two PDR genes appears in white. Shared neighborhoods are represented by colored connectors. A large connector links PDR genes sharing more than one common neighbor while a thin connector links PDR genes sharing one neighbor only. The numbers shown in blue is the number of the pillar in which the PDR gene has been classified on the Yeast Gene Order Browser [30]. The number shown in red is the number of the orthogroup in which the PDR gene has been classified by Synergy [46]. Click here for file
