# Supplementary material for: Combined phylogeny and neighborhood analysis of the evolution of the ABC transporters conferring multiple drug resistance in hemiascomycete yeasts
Source: BMC Genomics. 2009 Oct 1;10:459. doi: 10.1186/1471-2164-10-459 (PMC2763886; doi:10.1186/1471-2164-10-459)
Supplement: Additional file 1 — List of Pdrp features included in this paper. Sequences of S. cerevisiae [12], C. glabrata, K. lactis, D. hansenii, Y. lipolytica [54] and E. gossypii [55] were previously published. Z. rouxii and K. thermotolerans have both been sequenced by Génoscope, and S. kluyveri was sequenced by the Washington University Genome Sequencing Centre. The three new genomes ZYRO, KLTH, SAKL, plus KLLA have been entirely annotated and manually curated by the Génolevures consortium [29]. ERGO annotation is by GATTIKER[56]. [file 1471-2164-10-459-S1.PDF]

GL3 name	Abbreviated name	Subtelomeric	Phylogenetic cluster	SONS	Length	N-terminal Walker A	C-terminal ABC sig.
A. Homologs of Pdr5, Pdr10, Pdr12, Pdr15,SNQ2 and YNR070w							
SACE0P04972g	PDR12		A	a	1511	VGRPGAGCSTFL	NVEQRKKLSIGV
CAGL0M07293g	CAGL M07293		A	a	1515	VGRPGAGCSTLL	NVEQRKKLSIGV
ZYRO0F08866g	ZYRO F08866		A	a	1520	VGRPGAGSSTLL	NVEQRKKLSIGV
ZYRO0F08888g	ZYRO F08888		A	a	1520	VGRPGAGCSTLL	NVEQRKKLSIGV
SAKL0C05654g	SAKL C05654		A	a	1509	VGRPGAGCSTLL	NVEQRKKLSIGV
SAKL0H10670g	SAKL H10670		A	a	1509	VGRPGAGCSTLL	NVEQRKKLSIGV
KLLA0B09702g	KLLA B09702		A		1518	VGRPGAGCSTLL	NVEQRKKLSIGV
YALI0F17996g	YALI F17996		A		1508	LGRPGSGCSTFL	NVEQRKKLSIGV
SACE0N08954g	YNR070W	St	B		1333	LGRPGAGCTSFL	NVEQRKKLSIGV
SACE0D05478g	SNQ2		B	b	1501	LGRPGAGCSSFL	NVEQRKKLSIGV
CAGL0I04862g	CAGL I04862		B	b	1507	LGRPGAGCSSML	NVEQRKKLSIGV
ZYRO0B14762g	ZYRO B14762		B		1515	LGRPGSGCSSFL	NVEQRKKLSIGV
ZYRO0A04114g	ZYRO A04114		B	b	1518	LGRPGSGCSSFL	NVEQRKKLSIGV
KLTH0A01914g	KLTH A01914		B	b	1499	LGRPGAGCSSFL	NVEQRKKVSIGV
SAKL0H21010g	SAKL H21010		B		1447	LGRPGAGCSSFL	NVEQRKKLSIGV
SAKL0C11616g	SAKL C11616		B	b	1484	LGRPGAGCSSFL	NVEQRKKLSIGV
KLLA0D03432g	KLLA D03432		B	b	1483	LGRPGSGCSTFL	NVEQRKKLSIAV
ERGO0B08140g	ERGO B08140		B	b	1488	LGRPGAGCTSLL	NVEQRKKLSIGV
DEHA2A03696g	DEHA A03696		B		1477	LGRPGAGCSSLL	NVEQKKKLSIGV
SACE0O11000g	PDR10		C		1564	LGRPGAGCTTLL	NVEQRKRLTIGV
SACE0D14146g	PDR15		C	c	1529	LGRPGSGCTTLL	NVEQRKRLTIGV
SACE0O07216g	PDR5		C	c	1511	LGRPGSGCTTLL	NVEQRKRLTIGV
CAGL0M01760g	CAGL M01760		C		1499	LGRPGSGCTTLL	NVEQRKRLTIGV
CAGL0F02717g	CAGL F02717		C	c	1542	LGRPGSGCTTLL	NVEQRKRLTIGV
ZYRO0D17710g	ZYRO D17710	St	C		1486	LGRPGSGCTTLL	NVEQRKRLTIGV
ZYRO0D11836g	ZYRO D11836		C	c	1462	LGRPGSGCTTLL	NVEQRKRLTIGV
ZYRO0D11858g	ZYRO D11858		C	c	1503	LGRPGSGCTTLL	NVEQRKRLTIGV
ZYRO0D11880g	ZYRO D11880		C	c	1498	LGRPGSGCTTLL	NVEQRKRLTIGV
KLTH0G19448g	KLTH G19448		C	g	1486	LGRPGSGCSTLL	NVEQRKRLTVGV
SAKL0C06996g	SAKL C06996		C		1545	LGRPGAGCSTLL	NVEQRKRLTIGV
SAKL0C11704g	SAKL C11704		C	b	1510	LGRPGAGCSTLL	NVEQRKRLTIGV
SAKL0G08008g	SAKL G08008		C	f	1549	LGRPGSGCSTLL	NVEQRKRLTIGV
KLLA0D03476g	KLLA D03476		C	b	1560	LGRPGAGCSTLL	NVEQRKRLTIGV
KLLA0F21692g	KLLA F21692		C	f	1525	LGRPGSGCTTLL	NVEQRKRLTIGV
ERGO0G05126g	ERGO G05126		C		1497	LGRPGAGCSTLL	NVEQRKRLTIGV
ERGO0B08162g	ERGO B08162		C	b	1512	LGRPGAGCSTLL	NVEQRKRLTIGV
DEHA2B16610g	DEHA B16610	St	C		1508	LGRPGSGCSTLL	NVEQRKRLTIGV
DEHA2F16478g	DEHA F16478		C		1500	LGRPGSGCSTLL	NVEQRKRLTIGV
DEHA2G14894g	DEHA G14894		C		1481	LGRPGAGCSTFL	NVEQRKRLTIGV
DEHA2G14916g	DEHA G14916		C		1463	LGRPGAGCSTFL	NVEQRKRLTIGV
YALI0B02544g	YALI B02544		C		1485	LGRPGSGCTTLL	NVEQRKRLTIGV
YALI0B12980g	YALI B12980		C		1472	LGRPGSGCTTFL	NVEQRKRLTIGV
YALI0C20265g	YALI C20265		C		1469	LGRPGSGCTTFL	NVEQRKRLTIGV
YALI0E14729g	YALI E14729		C		1659	LGRPGSGCTTFL	NVEQRKRLTIGV

B. Homologs of YOL075c

SACE0O02112g	YOL075C		D	d	1294	MGGSGSGKTTLL	SGGEKRRVTMGV
CAGL0I08019g	CAGL I08019		D	d	1285	MGGSGSGKTTLL	SGGEKRRVSMGI
ZYRO0B05588g	ZYRO B05588		D	d	1270	MGGSGSGKTTLL	SGGEKRRVTIGV
KLTH0E15796g	KLTH E15796		D	d	1247	VGGSGSGKTTLL	SGGEKRRVSMGT
SAKL0C08074g	SAKL C08074		D	d	1257	VGGSGSGKTTLL	SGGEKRRVSLGV
KLLA0C04477g	KLLA C04477		D	d	1253	IGGSGSGKTTML	SGGEKRRVSIGI
YALI0D25828g	YALI D25828		D		1328	LGGSGSGKTSLL	SGGEKRRVSICV

C. Homologs of AUS1 and Pdr11

SACE0I03608g	PDR11		E		1411	LGNP----TSAL	SPTQRKLLSIGV
SACE0O04180g	AUS1		E	e	1394	LGYP----TSTL	NPTQRKLLSIGV
CAGL0F01419g	CAGL F01419		E	e	1398	LGAP----TSGI	NPTQRKLLSIGV
SAKL0F04312g	SAKL F04312		E		1338	NYKD----SSIL	NPAEKKLLSIGV
ERGO0F13266g	ERGO F13266		E		1309	LVVG----DKSS	CLQEKRILSIGP

D. Fragments and pseudogenes

KLTH0E17138g	KLTH E17138	St		g	405	/	-
KLTH0H00110g	KLTH H00110	St		g	660	LGRPGSGCTTLL	
SAKL0D15356g	SAKL D15356	St			72		
DEHA2C03784g	DEHA C03784				626	/	-
DEHA2F00154g	DEHA F00154	St			1099	-	NVEQRKRLTIGV
DEHA2F16500g	DEHA F16500				79	-	/
