# Supplementary material for: Combined phylogeny and neighborhood analysis of the evolution of the ABC transporters conferring multiple drug resistance in hemiascomycete yeasts
Source: BMC Genomics. 2009 Oct 1;10:459. doi: 10.1186/1471-2164-10-459 (PMC2763886; doi:10.1186/1471-2164-10-459)
Supplement: Additional file 2 — Schema of analysis tools and databases. Rectangles represent databases and results while parallelograms represent tools used to obtain them. [file 1471-2164-10-459-S2.PPT]

## Slide 1
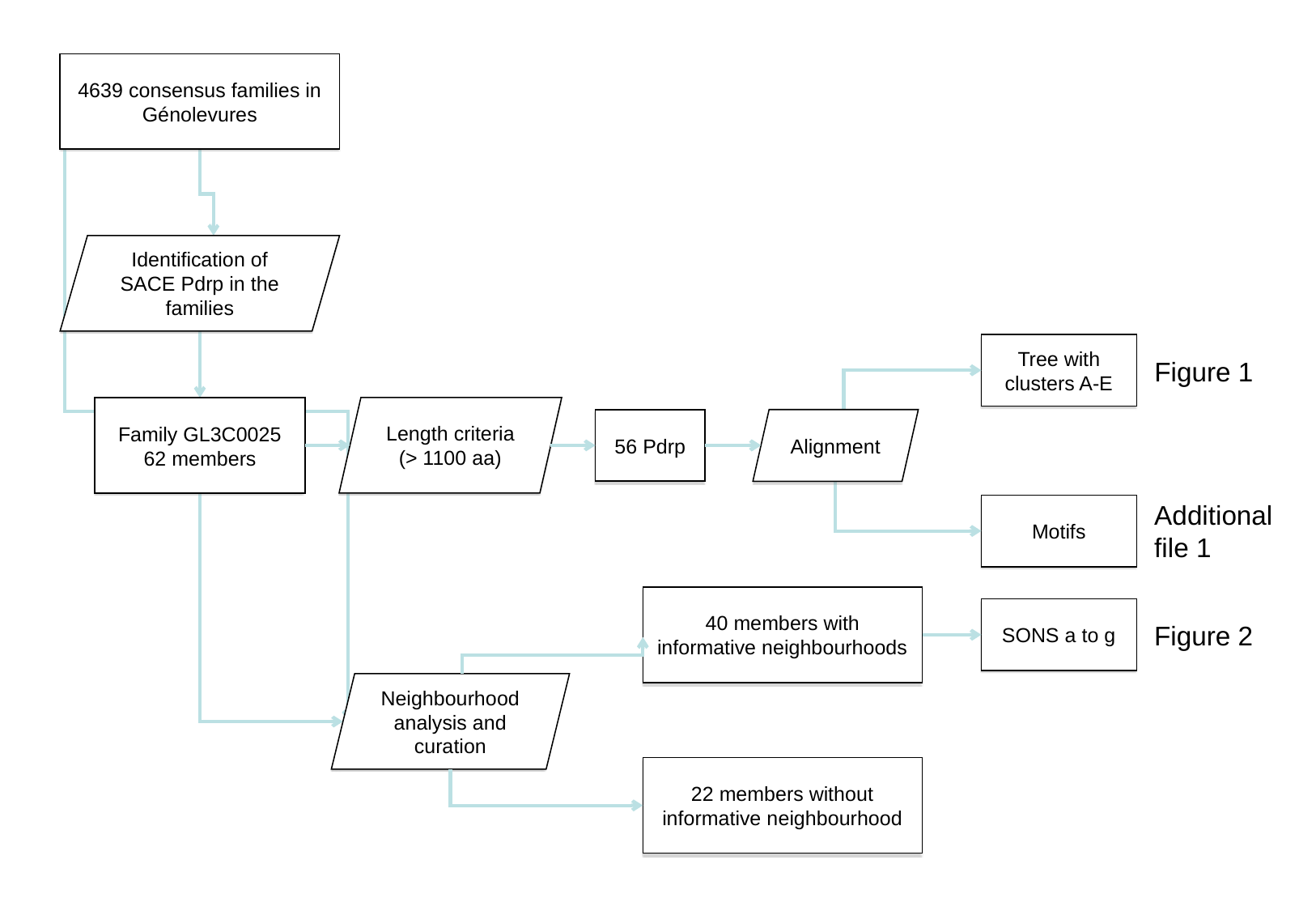

4639 consensus families in Génolevures
Identification of
SACE Pdrp in the families
Tree with clusters A-E
Figure 1
Length criteria
(> 1100 aa)
Family GL3C0025
62 members
56 Pdrp
Alignment
Additional file 1
Motifs
40 members with informative neighbourhoods
SONS a to g
Figure 2
Neighbourhood analysis and curation
22 members without informative neighbourhood
