# Supplementary material for: Combined phylogeny and neighborhood analysis of the evolution of the ABC transporters conferring multiple drug resistance in hemiascomycete yeasts
Source: BMC Genomics. 2009 Oct 1;10:459. doi: 10.1186/1471-2164-10-459 (PMC2763886; doi:10.1186/1471-2164-10-459)
Supplement: Additional file 3 — Steps of the Identification of Orthologs by Neighborhood and Similarity method. (1) The building of SONS (Subset of Orthologs defined by Neighborhood and Similarity) begins by taking a family of genes whose translation products show sequence similarity for example, the family GL3C0025. (2) For each query gene of this family we identify 15 genes, which we call neighbors, on each side and especially the family to which these neighbors belong. (3) Two query genes whose translation products belong to the same family (homologs by similarity) are members of the same SONS if they share at least one pair of neighbors that are also homologous to each other by similarity. These neighbors are highlighted in the same color. In the example, YOL075c shares some neighbors with the query gene of CAGL and has one neighbor in common with the query gene of KLTH. CAGL has no gene in common with KLTH, however YOL075c must be in the same SONS as the query genes of CAGL and KLTH. In consequence, the three query genes are clustered in the same SONS. Homologs that do not share a pair of homologous neighbors are separated into two distinct SONS. (4) See Figure 2 SONS d for the complete SONS of YOL075c. [file 1471-2164-10-459-S3.PPT]

## Slide 1
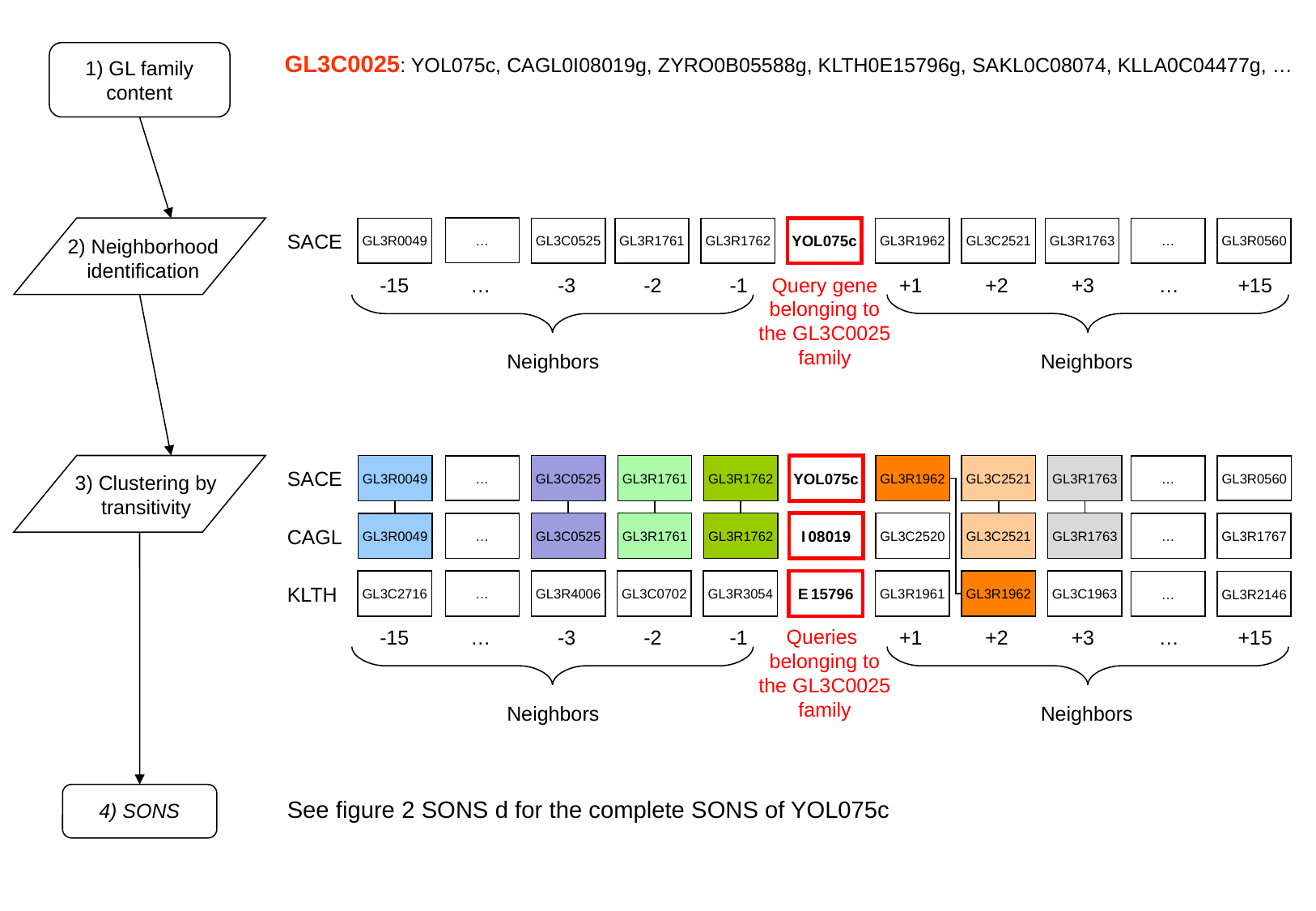

1) GL family content
GL3C0025: YOL075c, CAGL0I08019g, ZYRO0B05588g, KLTH0E15796g, SAKL0C08074, KLLA0C04477g, …
GL3R0049
…
GL3C0525
GL3R1761
GL3R1762
YOL075c
GL3R1962
GL3C2521
GL3R1763
…
GL3R0560
SACE
-15
…
-3
-2
-1
Query gene belonging to the GL3C0025
family
+1
+2
+3
…
+15
Neighbors
Neighbors
2) Neighborhood identification
GL3R0049
…
…
…
GL3C0525
GL3R1761
GL3R1762
YOL075c
GL3R1962
GL3C2521
GL3R1763
GL3R0560
…
SACE
GL3C0525
GL3R1761
GL3R1762
I 08019
GL3C2520
GL3C2521
GL3R1763
GL3R0049
…
GL3R1767
CAGL
GL3C2716
GL3R4006
GL3C0702
GL3R3054
E 15796
GL3R1961
GL3R1962
GL3C1963
…
GL3R2146
KLTH
-15
…
-3
-2
-1
Queries belonging to the GL3C0025
family
+1
+2
+3
…
+15
Neighbors
Neighbors
3) Clustering by transitivity
4) SONS
See figure 2 SONS d for the complete SONS of YOL075c
