# Supplementary figures and images for: Combined phylogeny and neighborhood analysis of the evolution of the ABC transporters conferring multiple drug resistance in hemiascomycete yeasts
Source: BMC Genomics. 2009 Oct 1;10:459. doi: 10.1186/1471-2164-10-459 (PMC2763886; doi:10.1186/1471-2164-10-459)

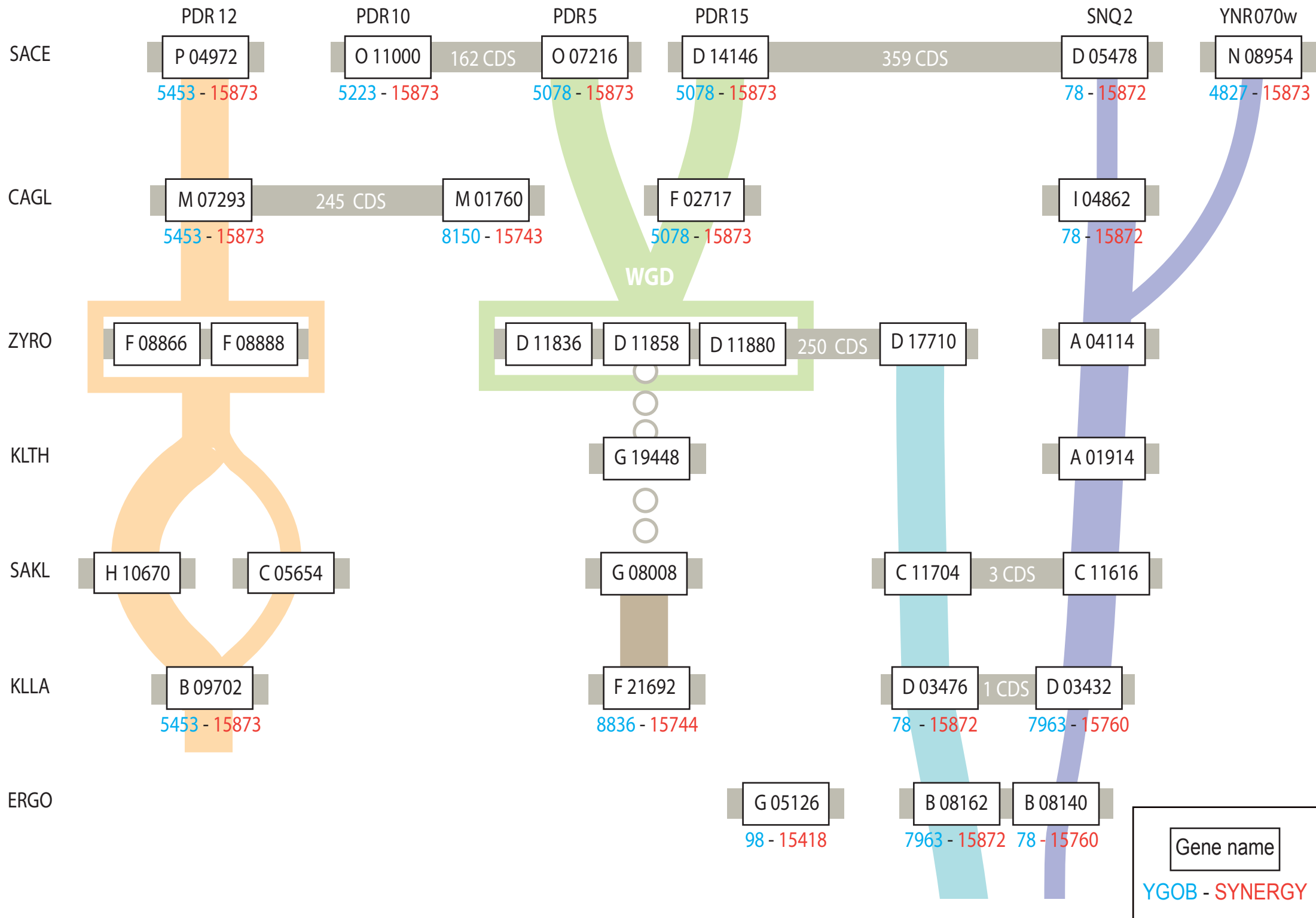

Supplement: Additional file 4 — Relationships between the Pdrp sensu stricto based on the neighborhood analysis: comparison with YGOB and SYNERGY. Each box represents a PDR. Grey lines join PDR genes located on the same chromosome. When non-adjacent, the number of coding sequences between two PDR genes appears in white. Shared neighborhoods are represented by colored connectors. A large connector links PDR genes sharing more than one common neighbor while a thin connector links PDR genes sharing one neighbor only. The numbers shown in blue is the number of the pillar in which the PDR gene has been classified on the Yeast Gene Order Browser [30]. The number shown in red is the number of the orthogroup in which the PDR gene has been classified by Synergy [46]. [file 1471-2164-10-459-S4.PDF]
